# Advances in Poultry Science: Nutritional Aspects of Poultry Production

**DOI:** 10.3390/vetsci13090888

**Published:** 2026-08-31

**Authors:** Catarina Stefanello, Valéria Biselo, Eduarda Alves Taschetto, Marco Antonio Ebbing

**Affiliations:** 1 Department of Animal Science, Federal University of Santa Maria, Santa Maria 97105-900, RS, Brazil; valeria.biselo@acad.ufsm.br (V.B.); taschetto.eduarda@acad.ufsm.br (E.A.T.); 2 Department of Animal Nutrition, Seara, Itajaí 88316-003, SC, Brazil; marcoantonioebbing@gmail.com

## Abstract

Poultry nutrition has evolved beyond simply formulating diets to meet nutrient requirements, with greater attention given to feed efficiency, animal health, environmental challenges, and the quality of poultry products. This Special Issue brings together 11 studies covering a range of topics, including alternative ingredients, functional dietary additives, feed processing, heat-stress mitigation, intestinal microbiota, and poultry product quality. The studies investigated insect meals, *Bombyx mori*, *Camelina sativa*, and *Spirulina platensis*, in addition to phytogenic compounds, selenium, fermented feeds, and enzyme-based approaches. The results show that these nutritional strategies can influence growth performance, nutrient utilization, immune and antioxidant responses, as well as meat and egg characteristics. At the same time, several factors may limit their application in commercial poultry production. These include differences in the composition of alternative ingredients, variation in responses under different experimental conditions, and the relatively small number of studies carried out under commercial production settings. Future studies should place greater emphasis on understanding their mechanisms of action, establishing appropriate inclusion levels, and assessing potential interactions among different strategies. It will also be important to consider the economic feasibility and environmental consequences of their use. Such information will help translate experimental findings into practical and more consistent nutritional strategies for poultry.

## 1. Editorial

Poultry nutrition is one of the most dynamic sectors of animal production, driven by the need to produce high-quality animal protein efficiently while addressing increasingly complex challenges related to feed costs, resource availability, environmental sustainability, animal health, welfare, and food safety. Within this context, current nutritional strategies increasingly consider nutrient utilization together with intestinal health, immune function, resilience to environmental stressors, and product quality. The Special Issue “Advances in Poultry Science: Nutritional Aspects of Poultry Production” in *Veterinary Sciences* brings together 11 contributions that reflect this broader perspective and highlight several current directions in poultry nutrition research.

One of the themes addressed in this Special Issue is the search for alternative and functional feed resources. Increasing pressure on conventional protein and energy sources has encouraged the evaluation of novel feed ingredients that could contribute to more resilient and sustainable poultry production systems. Singh et al. evaluated *Bombyx mori* meal and different lines of *Camelina sativa* using an in vitro digestibility approach. Their results indicate potential for these ingredients while pointing to an important methodological limitation. In vitro methods can be useful for screening individual feed ingredients, but they cannot yet fully replace in vivo digestibility studies of complete diets. This distinction is relevant, as poultry nutrition experts seek methods that can reduce the use of experimental animals without compromising the quality of nutritional evaluation.

Insect-derived products are another area of growing interest. The systematic review and meta-analysis by Dalmoro et al. synthesized evidence on *Hermetia illucens* and *Tenebrio molitor*, showing that insect meals and oils can provide nutrients as well as biologically active compounds, including chitin, lauric acid, and antimicrobial peptides. The analysis indicated that low levels of insect meal can be included in broiler diets without detrimental effects on performance. Potential effects on the intestinal microbiota and immune responses also suggest that alternative ingredients may have functional roles in addition to their nutritional value. However, variability in insect species, rearing substrates, processing methods, nutrient composition, and inclusion levels remains a major challenge for their wider adoption.

The contributions to this Special Issue also show that sustainability cannot be considered separately from feed quality and safety. Vidal et al. conducted a three-year evaluation of corn varieties carrying different transgenic events and found substantial differences in agronomic characteristics, nutrient composition, amino acid availability, energy value, and mycotoxin occurrence. These results show that the nutritional value of corn cannot be inferred from its botanical identity alone. Genetic background, environmental conditions, agronomic management, and contamination risks may all contribute to its nutritional value for poultry. Careful ingredient characterization and quality control are therefore important elements for feed formulation.

Another major topic addressed was the use of functional additives to support poultry health and performance. Two contributions investigated phytogenic compounds, microalgae, garlic, essential oils, and other zootechnical additives. Abdel-Wareth and Lohakare reported beneficial effects of oregano and peppermint bioactive lipid compounds on growth performance, blood biochemical parameters, and meat quality in broilers raised under hot climatic conditions. Attia et al. found that supplementation with *Spirulina platensis* and *Allium sativum* could alleviate several effects of heat stress, including changes in oxidative status, intestinal morphology, and intestinal microbial populations. Together, these studies support the potential use of phytogenic and other natural bioactive compounds as part of nutritional strategies designed to improve the ability of poultry to cope with environmental challenges.

The potential of *Spirulina platensis* was also examined in laying hens. Salahuddin et al. reported improvements in indicators of egg production and quality, including egg weight, egg mass, yolk color, Haugh units, and the maintenance of egg quality during storage. In addition to its nutritional value, this study highlights the possibility of using functional ingredients to modify characteristics of animal-derived foods that are relevant to consumers and food systems.

Heat stress is an important challenge for poultry production and is likely to become even more relevant under changing climatic conditions. Mahmoud et al. investigated nano-selenium in growing quails exposed to heat stress and reported improvements in growth performance and oxidative status, particularly at 0.2 mg/kg. The authors also found effects on the expression of genes related to antioxidant and inflammatory responses. Along with the studies involving *Spirulina*, garlic, oregano, and peppermint, these results support how diets can help poultry to support environmental stressors. Nevertheless, future research should go beyond determining whether a given additive improves performance during stress. Understanding the mechanisms underlying these responses, identifying which birds are most responsive, and determining how nutritional interventions interact with environmental conditions will be important for translating these findings into practical applications.

The interaction among feed processing, exogenous enzymes, nutrient utilization, and intestinal function represents another relevant research area in this Special Issue. Ibrahim et al. showed that solid-state fermentation of barley combined with fibrolytic exogenous enzymes improved nutrient utilization and antioxidant status and affected the expression of genes related to intestinal barrier function and nutrient transport. Their publication illustrates how feed processing and enzyme supplementation can be combined to improve the nutritional value of alternative cereals and address some of the limitations associated with non-starch polysaccharides. These results suggest that nutritional strategies should consider the feed matrix, processing conditions, enzyme activity, and host response as interconnected factors rather than as independent variables.

The relationship between diets and the intestinal microbiota was further examined by Popov et al., using a validated in vitro cecal model to investigate the effects of a phytogenic blend on microbial communities. The blend increased bacteria associated with energy metabolism and production performance while reducing potentially opportunistic bacteria. The study also highlights both the usefulness and the limitations of advanced in vitro models. Such systems can provide mechanistic information and facilitate preliminary screening, but their results require confirmation under in vivo conditions because in vivo models cannot reproduce the full physiological complexity of a living bird.

The study by Castro et al. demonstrated the complexity of evaluating feed additives, particularly when different products are used in combination. The combination of zootechnical additives with antimicrobial growth promoters improved performance and immune-related parameters, with essential oils showing positive effects. However, some combinations were associated with less favorable intestinal histomorphometric responses. These results are relevant because improvements in growth performance should not automatically be interpreted as evidence of improved intestinal health. Comprehensive evaluation of nutritional strategies should therefore include multiple complementary endpoints rather than relying solely on growth performance or feed conversion.

Finally, the Special Issue highlights the increasing contribution of advanced analytical approaches to poultry science. Miao et al. used non-targeted metabolomics combined with a random forest algorithm to characterize changes in small-molecule metabolites in refrigerated goose breast meat. The identification of metabolites associated with the degradation of carbohydrates, amino acids, nucleotides, and lipids provides a more detailed view of the processes involved in meat deterioration and may help identify biomarkers for monitoring freshness. Although this was not a conventional feed trial, the study broadens the scope of poultry science by showing how modern analytical approaches can link metabolism, product quality, and food safety. Such approaches may also provide opportunities to better connect nutritional interventions with downstream changes in animal metabolism and product characteristics.

## 2. Remaining Knowledge Gaps

Taken together, the contributions to this Special Issue demonstrate substantial progress in poultry nutrition while also pointing to important knowledge gaps. One of the main challenges is that the efficacy of alternative ingredients and functional additives remains highly context-dependent. Ingredient origin, chemical composition, processing, inclusion level, bird genotype and age, health status, environmental conditions, and basal diet can all influence the magnitude and direction of the response. Positive results obtained under specific experimental conditions should therefore be interpreted with caution and cannot, by themselves, support universal nutritional recommendations.

Another area that deserves greater attention is the identification of mechanisms of action. Nutritional interventions are often assessed primarily through growth performance, blood biochemical parameters, intestinal morphology, or selected immune indicators. Although these measurements provide important information, they do not necessarily reveal the biological pathways underlying the observed responses. The integration of microbiome, metabolome, transcriptome, proteome, and physiological data could provide a more comprehensive understanding of how dietary components interact with the host and, importantly, help distinguish associations from potential causal mechanisms.

Translating experimental findings into commercial practice is another persistent challenge. Many studies are conducted under controlled conditions and over relatively short experimental periods, whereas commercial poultry production is characterized by changing environmental conditions, variation in ingredient quality, vaccination programs, disease pressure, stocking density, feed manufacturing conditions, and multiple concurrent stressors. Nutritional strategies therefore need to be tested under conditions that more closely represent the complexity and variability of commercial production.

Sustainability also deserves a broader and more consistent definition. The term is increasingly used in research on alternative ingredients and feed additives, but nutritional sustainability encompasses environmental, economic, biological, and social dimensions. An ingredient should not be considered sustainable simply because it is novel or locally available. Future evaluations should consider factors such as life-cycle impacts, land and water use, greenhouse gas emissions, feed cost, scalability, competition for resources, and consistency of supply. A more comprehensive assessment of these factors will be essential to determining whether nutritional innovations can provide practical and lasting benefits to poultry production.

## 3. Future Perspectives

The next phase of poultry nutrition research can move toward more integrated and precision-oriented approaches. Rather than focusing only on whether a particular ingredient or additive improves body weight or feed conversion, future studies should examine how nutrition can simultaneously influence nutrient utilization, intestinal function, immune competence, resilience to environmental stress, animal welfare, product quality, and environmental performance.

Precision nutrition represents an important direction in this context. Advances in analytical technologies and computational modeling may allow diets to be formulated using more accurate estimates of nutrient digestibility, bird requirements, environmental conditions, and variability among ingredients, birds or flocks. Such approaches could help reduce nutrient over-supply and improve feed efficiency while limiting losses of nitrogen, phosphorus, and other nutrients.

The integration of microbiome and metabolomics research is also likely to become important. Identifying not only which microorganisms are present but also the metabolites they produce may provide a better understanding of how dietary fiber, enzymes, phytogenics, organic acids, probiotics, prebiotics, and alternative ingredients influence bird health. Metabolomics, as illustrated by the study on goose meat, may also help identify biomarkers that link metabolic status with product quality and potentially provide new tools for monitoring nutritional responses.

Another priority is the rational combination of nutritional strategies. The poultry industry increasingly uses combinations of enzymes, phytogenics, organic acids, probiotics, prebiotics, minerals, and alternative ingredients, yet the additive, synergistic, or antagonistic interactions among these components are still not well understood. Experimental designs based on complementary mechanisms, rather than combining several additives in the same treatment, could provide more useful information for practical application.

Interactions among exogenous enzymes provide a good example of this complexity. The effects of xylanase and phytase on nutrient utilization may depend on diet composition, with phytase potentially modifying the response to xylanase in terms of energy and protein digestibility. Stefanello et al. [1] reported complementary effects of exogenous carbohydrases, with the combination of amylase and xylanase increasing metabolizable energy by 2.5% and starch digestibility by 3% compared with non-supplemented diets. These findings suggest that combining enzymes with complementary modes of action may improve nutrient and energy utilization in broilers, although responses are likely to depend on the characteristics of the basal diet and the specific enzymes used.

The combination of organic acids and probiotics provides another example. Rodjan et al. [2] reported no improvement in broiler performance when these additives were combined, but observed increases in crude fiber digestibility and duodenal villus height, together with a reduction in *Escherichia coli* populations. Thus, a combined intervention may influence intestinal health without necessarily resulting in an additional improvement in growth performance. Similarly, combinations of organic acids and essential oils with dietary fiber have been investigated as strategies to simultaneously influence intestinal morphology, microbiota, and fermentation, although responses may vary according to diet composition and feed additives [3]. The magnitude and direction of the effects of combined nutritional interventions may depend on dietary composition, ingredient characteristics, enzyme dose, additive type and dose, bird age, and the biological endpoint being evaluated.

Equally important is the development of standardized approaches for evaluating novel feed ingredients and additives. This should include consistent reporting of ingredient composition, processing methods, bioactive compounds, experimental conditions, and biological endpoints. Greater standardization would facilitate comparisons among studies and strengthen the reliability of meta-analyses and evidence-based nutritional recommendations.

Climate resilience should also become a central component of nutritional research. Heat stress is likely to become increasingly important as global temperatures rise. Nutritional interventions that support antioxidant defenses, intestinal integrity, immune function, and feed efficiency under heat stress may contribute to more resilient production systems. Future studies should consider not only acute experimental heat stress but also chronic and cyclic thermal challenges that reflect commercial production environments.

Finally, experimental nutrition needs to be increasingly connected with real-world sustainability. Alternative feed ingredients, feed processing technologies, and functional additives should be assessed not only for their biological efficacy but also for economic feasibility, scalability, availability, environmental footprint, and consistency under commercial conditions. Addressing these questions will require multidisciplinary collaboration among animal nutritionists, veterinarians, microbiologists, food scientists, environmental scientists, economists, and data scientists. Such collaboration will be essential to translating nutritional innovations into strategies that are biologically effective, economically viable, and environmentally responsible.

## 4. Conclusions

The 11 contributions included in this Special Issue reflect the breadth and rapid evolution of poultry nutrition research. They address the potential of alternative feed resources, phytogenic compounds, microalgae, insect-derived products, feed fermentation, exogenous enzymes, mineral technologies, microbiota-targeted interventions, and advanced analytical approaches to support more efficient poultry production. At the same time, the studies show that nutritional responses are multifactorial and cannot be adequately evaluated through performance measurements alone.

The future of poultry nutrition will depend on a more integrated view of nutrient utilization, intestinal health, immunity, microbiota, metabolism, environmental resilience, product quality, economics, and sustainability. Continued progress will require rigorous mechanistic research, standardized methodologies, validation under commercial conditions, and the effective use of emerging analytical and computational tools. Ultimately, the value of future nutritional innovations will depend not only on their ability to improve biological performance but also on whether they can be translated into practical, economically viable, and environmentally responsible solutions for a growing poultry industry.

## Data Availability

No new data were created.
